# *N*′-(4-Diethyl­amino-2-hy­droxy­benzyl­idene)-4-(dimethyl­amino)­benzo­hydrazide methanol monosolvate

**DOI:** 10.1107/S1600536811019957

**Published:** 2011-06-11

**Authors:** Fu Su, Zheng-Gui Gu, Jun Lin

**Affiliations:** a Jiangsu Centre of Extraction Separation Engineering Technology, College of Chemistry and Materials Science, Nanjing Normal University, Nanjing 210046, People’s Republic of China

## Abstract

The title compound, C_20_H_26_N_4_O_2_·CH_3_OH, was prepared by the reaction of 4-diethyl­amino-2-hy­droxy­benzaldehyde with 4-(dimethyl­amino)­benzohydrazide. The dihedral angle between the two benzene rings is 13.6 (3)° and an intra­molecular O—H⋯N hydrogen bond generates an *S*(6) ring. In the crystal, the hydrazone and methanol mol­ecules are linked through inter­molecular O—H⋯O and N—H⋯O hydrogen bonds, forming chains along *a*.

## Related literature

For the biological properties of hydrazones, see: Ajani *et al.* (2010 ▶); Zhang *et al.* (2010 ▶); Angelusiu *et al.* (2010 ▶). For similar structures, see: Huang & Wu (2010 ▶); Khaledi *et al.* (2010 ▶); Zhou & Yang (2010 ▶); Ji & Lu (2010 ▶); Singh & Singh (2010 ▶); Ahmad *et al.* (2010 ▶). For hydrogen-bond motifs, see Bernstein *et al.* (1995 ▶).
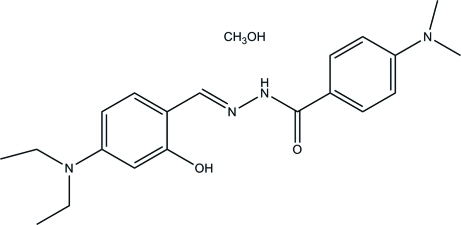


## Experimental

### 

#### Crystal data

C_20_H_26_N_4_O_2_·CH_4_O
*M* *_r_* = 386.49
Triclinic, 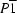

*a* = 6.786 (3) Å
*b* = 11.791 (3) Å
*c* = 14.252 (2) Å
α = 111.511 (3)°
β = 92.811 (2)°
γ = 96.492 (2)°
*V* = 1049.1 (6) Å^3^
*Z* = 2
Mo *K*α radiation
μ = 0.08 mm^−1^
*T* = 298 K
0.27 × 0.23 × 0.21 mm

#### Data collection

Bruker SMART CCD area-detector diffractometer
Absorption correction: multi-scan (*SADABS*; Bruker, 2001 ▶) *T*_min_ = 0.978, *T*_max_ = 0.983
7543 measured reflections
4421 independent reflections
1809 reflections with *I* > 2σ(*I*)
*R*_int_ = 0.045

#### Refinement

*R*[*F* ^2^ > 2σ(*F* ^2^)] = 0.070
*wR*(*F* ^2^) = 0.189
*S* = 1.00
4421 reflections
263 parameters
1 restraint
H atoms treated by a mixture of independent and constrained refinement
Δρ_max_ = 0.30 e Å^−3^
Δρ_min_ = −0.21 e Å^−3^

### 

Data collection: *SMART* (Bruker, 2007 ▶); cell refinement: *SAINT* (Bruker, 2007 ▶); data reduction: *SAINT*; program(s) used to solve structure: *SHELXTL* (Sheldrick, 2008 ▶); program(s) used to refine structure: *SHELXTL*; molecular graphics: *SHELXTL*; software used to prepare material for publication: *SHELXTL*.

## Supplementary Material

Crystal structure: contains datablock(s) global, I. DOI: 10.1107/S1600536811019957/sj5154sup1.cif

Structure factors: contains datablock(s) I. DOI: 10.1107/S1600536811019957/sj5154Isup2.hkl

Supplementary material file. DOI: 10.1107/S1600536811019957/sj5154Isup3.cml

Additional supplementary materials:  crystallographic information; 3D view; checkCIF report

## Figures and Tables

**Table 1:** Hydrogen-bond geometry (Å, °)

*D*—H⋯*A*	*D*—H	H⋯*A*	*D*⋯*A*	*D*—H⋯*A*
N2—H2⋯O3	0.90 (1)	2.07 (2)	2.936 (4)	160 (3)
O3—H3⋯O2^i^	0.82	1.84	2.661 (3)	177
O1—H1⋯N1	0.82	2.02	2.727 (3)	145

Symmetry code: (i) 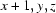.

**Fig. 1.**
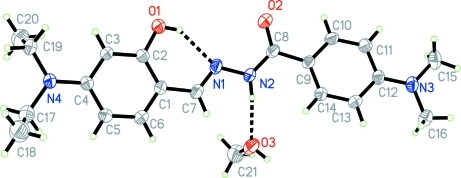
The molecular structure of the title compound showing 30% probability displacement ellipsoids and the atomic numbering. Hydrogen bonds are drawn as dashed lines.

**Fig. 2.**
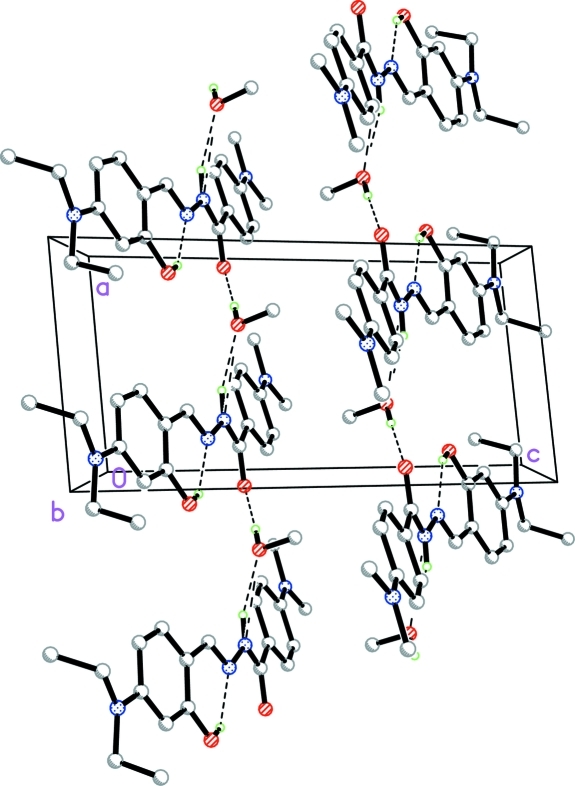
Crystal packing of the title compound, viewed down the b axis. Intermolecular interactions are drawn as dashed lines.

## supplementary crystallographic information

### Comment

In the last year, much attention has been focused on the biological properties of hydrazone compounds (Ajani *et al.*, 2010; Zhang *et al.*, 2010; Angelusiu *et al.*, 2010). The crystal structures of a number of hydrazone compounds have also been determined (Huang & Wu, 2010; Khaledi *et al.*, 2010; Zhou & Yang, 2010; Ji & Lu, 2010; Singh & Singh, 2010; Ahmad *et al.*, 2010). In this paper, the title new hydrazone compound, Fig. 1, is reported.

The asymmetric unit of the compound contains a hydrazone molecule and a methanol molecule. The dihedral angle between the two benzene rings is 13.6 (3)° and an intramolecular O1—H1···N1 hydrogen bond forms an S(6) ring (Bernstein *et al.*, 1995). In the crystal structure, the hydrazone and methanol molecules are linked through intermolecular O–H···O and N–H···O hydrogen bonds (Table 1), to form 1D chains along *a* (Fig. 2).

### Experimental

The reaction of 4-diethylamino-2-hydroxybenzaldehyde (0.193 g, 1 mmol) with 4-(dimethylamino)benzohydrazide (0.179 g, 1 mmol) in 30 ml methanol at room temperature afforded the title compound. Colorless single crystals were formed by gradual evaporation of the solution in air.

### Refinement

The amino H atom was located in a difference Fourier map and refined with the N–H distance restrained to be  0.90 (1) Å, and with *U*_iso_ = 0.08 Å^2^. The remaining H atoms were positioned geometrically (C–H = 0.93-0.97 Å, O–H = 0.82 Å) and refined as riding with *U*_iso_(H) = 1.2*U*_eq_(C) or 1.5 *U*_eq_(O3 and C_methyl_).

### Crystal data

**Table:**

C_20_H_26_N_4_O_2_·CH_4_O	*Z* = 2
*M**_r_* = 386.49	*F*(000) = 416
Triclinic, *P*1	*D*_x_ = 1.224 Mg m^−^^3^
Hall symbol: -P 1	Mo *K*α radiation, λ = 0.71073 Å
*a* = 6.786 (3) Å	Cell parameters from 765 reflections
*b* = 11.791 (3) Å	θ = 2.6–24.5°
*c* = 14.252 (2) Å	µ = 0.08 mm^−^^1^
α = 111.511 (3)°	*T* = 298 K
β = 92.811 (2)°	Block, colorless
γ = 96.492 (2)°	0.27 × 0.23 × 0.21 mm
*V* = 1049.1 (6) Å^3^	

### Data collection

**Table:**

Bruker SMART CCD area-detector diffractometer	4421 independent reflections
Radiation source: fine-focus sealed tube	1809 reflections with *I* > 2σ(*I*)
graphite	*R*_int_ = 0.045
ω scans	θ_max_ = 27.0°, θ_min_ = 2.8°
Absorption correction: multi-scan (*SADABS*; Bruker, 2001)	*h* = −8→8
*T*_min_ = 0.978, *T*_max_ = 0.983	*k* = −15→15
7543 measured reflections	*l* = −15→18

### Refinement

**Table:**

Refinement on *F*^2^	Primary atom site location: structure-invariant direct methods
Least-squares matrix: full	Secondary atom site location: difference Fourier map
*R*[*F*^2^ > 2σ(*F*^2^)] = 0.070	Hydrogen site location: inferred from neighbouring sites
*wR*(*F*^2^) = 0.189	H atoms treated by a mixture of independent and constrained refinement
*S* = 1.00	*w* = 1/[σ^2^(*F*_o_^2^) + (0.0676*P*)^2^] where *P* = (*F*_o_^2^ + 2*F*_c_^2^)/3
4421 reflections	(Δ/σ)_max_ < 0.001
263 parameters	Δρ_max_ = 0.30 e Å^−^^3^
1 restraint	Δρ_min_ = −0.21 e Å^−^^3^

### Special details

**Table:**

Geometry. All esds (except the esd in the dihedral angle between two l.s. planes) are estimated using the full covariance matrix. The cell esds are taken into account individually in the estimation of esds in distances, angles and torsion angles; correlations between esds in cell parameters are only used when they are defined by crystal symmetry. An approximate (isotropic) treatment of cell esds is used for estimating esds involving l.s. planes.
Refinement. Refinement of F^2^ against ALL reflections. The weighted R-factor wR and goodness of fit S are based on F^2^, conventional R-factors R are based on F, with F set to zero for negative F^2^. The threshold expression of F^2^ > 2sigma(F^2^) is used only for calculating R-factors(gt) etc. and is not relevant to the choice of reflections for refinement. R-factors based on F^2^ are statistically about twice as large as those based on F, and R- factors based on ALL data will be even larger.

### Fractional atomic coordinates and isotropic or equivalent isotropic displacement parameters (Å^2^)

**Table:**

	*x*	*y*	*z*	*U*_iso_*/*U*_eq_	
O1	−0.1048 (3)	0.5092 (2)	0.2045 (2)	0.0741 (8)	
H1	−0.0686	0.4564	0.2235	0.111*	
O2	−0.0566 (3)	0.2247 (2)	0.3184 (2)	0.0770 (8)	
O3	0.6751 (4)	0.3787 (2)	0.3427 (2)	0.0907 (9)	
H3	0.7586	0.3321	0.3371	0.136*	
N1	0.1680 (4)	0.3808 (2)	0.2520 (2)	0.0549 (7)	
N2	0.2452 (4)	0.2985 (2)	0.2885 (2)	0.0564 (8)	
N3	0.4202 (4)	−0.1790 (2)	0.3829 (2)	0.0632 (8)	
N4	0.1131 (4)	0.7762 (2)	0.0366 (2)	0.0705 (9)	
C1	0.2398 (4)	0.5230 (2)	0.1692 (2)	0.0488 (8)	
C2	0.0478 (5)	0.5553 (3)	0.1644 (2)	0.0487 (8)	
C3	0.0066 (4)	0.6387 (3)	0.1211 (2)	0.0535 (9)	
H3A	−0.1218	0.6588	0.1194	0.064*	
C4	0.1541 (5)	0.6933 (3)	0.0801 (2)	0.0525 (8)	
C5	0.3450 (5)	0.6589 (3)	0.0828 (3)	0.0649 (10)	
H5	0.4457	0.6922	0.0546	0.078*	
C6	0.3848 (5)	0.5769 (3)	0.1263 (3)	0.0594 (9)	
H6	0.5130	0.5564	0.1273	0.071*	
C7	0.2927 (5)	0.4383 (3)	0.2145 (2)	0.0523 (8)	
H7	0.4248	0.4246	0.2168	0.063*	
C8	0.1245 (5)	0.2195 (3)	0.3155 (2)	0.0522 (8)	
C9	0.2154 (4)	0.1219 (3)	0.3371 (2)	0.0470 (8)	
C10	0.0978 (5)	0.0474 (3)	0.3749 (3)	0.0573 (9)	
H10	−0.0301	0.0644	0.3897	0.069*	
C11	0.1637 (5)	−0.0504 (3)	0.3910 (2)	0.0596 (9)	
H11	0.0806	−0.0973	0.4173	0.071*	
C12	0.3538 (5)	−0.0806 (3)	0.3687 (2)	0.0505 (8)	
C13	0.4723 (4)	−0.0056 (3)	0.3301 (2)	0.0575 (9)	
H13	0.6001	−0.0224	0.3148	0.069*	
C14	0.4036 (5)	0.0927 (3)	0.3145 (2)	0.0553 (9)	
H14	0.4855	0.1402	0.2883	0.066*	
C15	0.2976 (6)	−0.2536 (3)	0.4256 (3)	0.0780 (11)	
H15A	0.2808	−0.2051	0.4946	0.117*	
H15B	0.3614	−0.3231	0.4233	0.117*	
H15C	0.1696	−0.2821	0.3869	0.117*	
C16	0.6191 (5)	−0.2071 (3)	0.3638 (3)	0.0789 (12)	
H16A	0.6412	−0.2160	0.2956	0.118*	
H16B	0.6347	−0.2824	0.3728	0.118*	
H16C	0.7139	−0.1414	0.4103	0.118*	
C17	0.2791 (6)	0.8609 (3)	0.0203 (3)	0.0792 (12)	
H17A	0.2346	0.9396	0.0307	0.095*	
H17B	0.3917	0.8751	0.0698	0.095*	
C18	0.3427 (6)	0.8094 (4)	−0.0821 (3)	0.1027 (14)	
H18A	0.3853	0.7310	−0.0929	0.154*	
H18B	0.4511	0.8645	−0.0893	0.154*	
H18C	0.2332	0.7989	−0.1312	0.154*	
C19	−0.0856 (5)	0.8062 (3)	0.0254 (3)	0.0603 (9)	
H19A	−0.1818	0.7332	0.0115	0.072*	
H19B	−0.0975	0.8310	−0.0322	0.072*	
C20	−0.1341 (6)	0.9075 (3)	0.1181 (3)	0.0841 (12)	
H20A	−0.1327	0.8810	0.1742	0.126*	
H20B	−0.2638	0.9272	0.1057	0.126*	
H20C	−0.0366	0.9791	0.1336	0.126*	
C21	0.7364 (6)	0.4885 (4)	0.4255 (3)	0.0977 (14)	
H21A	0.8506	0.5328	0.4108	0.146*	
H21B	0.7706	0.4704	0.4841	0.146*	
H21C	0.6301	0.5378	0.4384	0.146*	
H2	0.3791 (16)	0.309 (3)	0.290 (3)	0.080*	

### Atomic displacement parameters (Å^2^)

**Table:**

	*U*^11^	*U*^22^	*U*^33^	*U*^12^	*U*^13^	*U*^23^
O1	0.0555 (14)	0.0899 (18)	0.111 (2)	0.0186 (12)	0.0232 (14)	0.0730 (17)
O2	0.0539 (15)	0.0793 (16)	0.118 (2)	0.0240 (13)	0.0139 (14)	0.0555 (15)
O3	0.0596 (17)	0.0795 (18)	0.137 (3)	0.0214 (13)	−0.0033 (17)	0.0440 (18)
N1	0.0575 (18)	0.0537 (15)	0.062 (2)	0.0165 (13)	−0.0006 (15)	0.0301 (15)
N2	0.0473 (16)	0.0592 (16)	0.078 (2)	0.0149 (14)	0.0037 (16)	0.0420 (16)
N3	0.071 (2)	0.0561 (16)	0.077 (2)	0.0165 (15)	0.0121 (16)	0.0391 (16)
N4	0.0546 (18)	0.0831 (19)	0.108 (3)	0.0214 (15)	0.0239 (17)	0.070 (2)
C1	0.0493 (19)	0.0476 (17)	0.055 (2)	0.0121 (15)	0.0030 (16)	0.0239 (16)
C2	0.048 (2)	0.0527 (18)	0.051 (2)	0.0079 (15)	0.0085 (16)	0.0261 (17)
C3	0.0428 (19)	0.0593 (19)	0.071 (3)	0.0145 (16)	0.0099 (17)	0.0364 (18)
C4	0.050 (2)	0.0564 (18)	0.062 (2)	0.0109 (16)	0.0111 (17)	0.0329 (18)
C5	0.049 (2)	0.078 (2)	0.091 (3)	0.0170 (18)	0.0227 (19)	0.054 (2)
C6	0.047 (2)	0.064 (2)	0.078 (3)	0.0168 (16)	0.0093 (18)	0.036 (2)
C7	0.051 (2)	0.0497 (18)	0.058 (2)	0.0168 (16)	0.0002 (17)	0.0206 (17)
C8	0.046 (2)	0.0579 (19)	0.058 (2)	0.0172 (16)	0.0043 (17)	0.0248 (17)
C9	0.0447 (19)	0.0527 (18)	0.049 (2)	0.0133 (15)	0.0031 (16)	0.0233 (16)
C10	0.0446 (19)	0.071 (2)	0.070 (3)	0.0175 (16)	0.0158 (17)	0.038 (2)
C11	0.057 (2)	0.066 (2)	0.069 (3)	0.0114 (18)	0.0119 (19)	0.0401 (19)
C12	0.058 (2)	0.0480 (17)	0.050 (2)	0.0094 (16)	−0.0002 (17)	0.0233 (16)
C13	0.0447 (19)	0.061 (2)	0.075 (3)	0.0165 (16)	0.0112 (18)	0.0314 (19)
C14	0.050 (2)	0.0555 (18)	0.072 (3)	0.0133 (16)	0.0117 (18)	0.0353 (18)
C15	0.108 (3)	0.061 (2)	0.079 (3)	0.020 (2)	0.015 (2)	0.040 (2)
C16	0.071 (3)	0.069 (2)	0.109 (3)	0.0221 (19)	0.004 (2)	0.043 (2)
C17	0.079 (3)	0.083 (2)	0.094 (4)	0.025 (2)	0.017 (2)	0.050 (2)
C18	0.113 (4)	0.129 (4)	0.092 (4)	0.038 (3)	0.036 (3)	0.062 (3)
C19	0.058 (2)	0.066 (2)	0.069 (3)	0.0134 (17)	0.0033 (19)	0.038 (2)
C20	0.085 (3)	0.080 (3)	0.089 (3)	0.023 (2)	0.011 (2)	0.030 (2)
C21	0.101 (3)	0.090 (3)	0.114 (4)	0.029 (3)	0.024 (3)	0.046 (3)

### Geometric parameters (Å, °)

**Table:**

O1—C2	1.362 (3)	C10—C11	1.373 (4)
O1—H1	0.8200	C10—H10	0.9300
O2—C8	1.239 (3)	C11—C12	1.397 (4)
O3—C21	1.397 (4)	C11—H11	0.9300
O3—H3	0.8200	C12—C13	1.404 (4)
N1—C7	1.281 (4)	C13—C14	1.383 (4)
N1—N2	1.396 (3)	C13—H13	0.9300
N2—C8	1.344 (4)	C14—H14	0.9300
N2—H2	0.900 (10)	C15—H15A	0.9600
N3—C12	1.368 (3)	C15—H15B	0.9600
N3—C16	1.441 (4)	C15—H15C	0.9600
N3—C15	1.454 (4)	C16—H16A	0.9600
N4—C4	1.381 (4)	C16—H16B	0.9600
N4—C19	1.448 (4)	C16—H16C	0.9600
N4—C17	1.504 (4)	C17—C18	1.469 (5)
C1—C6	1.396 (4)	C17—H17A	0.9700
C1—C2	1.403 (4)	C17—H17B	0.9700
C1—C7	1.441 (4)	C18—H18A	0.9600
C2—C3	1.384 (4)	C18—H18B	0.9600
C3—C4	1.395 (4)	C18—H18C	0.9600
C3—H3A	0.9300	C19—C20	1.501 (5)
C4—C5	1.404 (4)	C19—H19A	0.9700
C5—C6	1.369 (4)	C19—H19B	0.9700
C5—H5	0.9300	C20—H20A	0.9600
C6—H6	0.9300	C20—H20B	0.9600
C7—H7	0.9300	C20—H20C	0.9600
C8—C9	1.484 (4)	C21—H21A	0.9600
C9—C14	1.382 (4)	C21—H21B	0.9600
C9—C10	1.389 (4)	C21—H21C	0.9600
			
C2—O1—H1	109.5	C14—C13—C12	121.6 (3)
C21—O3—H3	109.5	C14—C13—H13	119.2
C7—N1—N2	115.4 (3)	C12—C13—H13	119.2
C8—N2—N1	121.0 (3)	C9—C14—C13	121.3 (3)
C8—N2—H2	129 (2)	C9—C14—H14	119.4
N1—N2—H2	110 (2)	C13—C14—H14	119.4
C12—N3—C16	121.6 (3)	N3—C15—H15A	109.5
C12—N3—C15	121.0 (3)	N3—C15—H15B	109.5
C16—N3—C15	117.3 (3)	H15A—C15—H15B	109.5
C4—N4—C19	122.5 (3)	N3—C15—H15C	109.5
C4—N4—C17	120.5 (3)	H15A—C15—H15C	109.5
C19—N4—C17	115.6 (2)	H15B—C15—H15C	109.5
C6—C1—C2	116.7 (3)	N3—C16—H16A	109.5
C6—C1—C7	119.4 (3)	N3—C16—H16B	109.5
C2—C1—C7	123.9 (3)	H16A—C16—H16B	109.5
O1—C2—C3	116.9 (3)	N3—C16—H16C	109.5
O1—C2—C1	121.8 (3)	H16A—C16—H16C	109.5
C3—C2—C1	121.3 (3)	H16B—C16—H16C	109.5
C2—C3—C4	121.2 (3)	C18—C17—N4	111.6 (3)
C2—C3—H3A	119.4	C18—C17—H17A	109.3
C4—C3—H3A	119.4	N4—C17—H17A	109.3
N4—C4—C3	121.3 (3)	C18—C17—H17B	109.3
N4—C4—C5	121.2 (3)	N4—C17—H17B	109.3
C3—C4—C5	117.5 (3)	H17A—C17—H17B	108.0
C6—C5—C4	120.9 (3)	C17—C18—H18A	109.5
C6—C5—H5	119.6	C17—C18—H18B	109.5
C4—C5—H5	119.6	H18A—C18—H18B	109.5
C5—C6—C1	122.4 (3)	C17—C18—H18C	109.5
C5—C6—H6	118.8	H18A—C18—H18C	109.5
C1—C6—H6	118.8	H18B—C18—H18C	109.5
N1—C7—C1	123.7 (3)	N4—C19—C20	112.6 (3)
N1—C7—H7	118.2	N4—C19—H19A	109.1
C1—C7—H7	118.2	C20—C19—H19A	109.1
O2—C8—N2	121.6 (3)	N4—C19—H19B	109.1
O2—C8—C9	121.1 (3)	C20—C19—H19B	109.1
N2—C8—C9	117.2 (3)	H19A—C19—H19B	107.8
C14—C9—C10	117.2 (3)	C19—C20—H20A	109.5
C14—C9—C8	124.7 (3)	C19—C20—H20B	109.5
C10—C9—C8	117.9 (3)	H20A—C20—H20B	109.5
C11—C10—C9	122.3 (3)	C19—C20—H20C	109.5
C11—C10—H10	118.8	H20A—C20—H20C	109.5
C9—C10—H10	118.8	H20B—C20—H20C	109.5
C10—C11—C12	121.0 (3)	O3—C21—H21A	109.5
C10—C11—H11	119.5	O3—C21—H21B	109.5
C12—C11—H11	119.5	H21A—C21—H21B	109.5
N3—C12—C11	121.6 (3)	O3—C21—H21C	109.5
N3—C12—C13	121.7 (3)	H21A—C21—H21C	109.5
C11—C12—C13	116.7 (3)	H21B—C21—H21C	109.5

### Hydrogen-bond geometry (Å, °)

**Table:**

*D*—H···*A*	*D*—H	H···*A*	*D*···*A*	*D*—H···*A*
N2—H2···O3	0.90 (1)	2.07 (2)	2.936 (4)	160 (3)
O3—H3···O2^i^	0.82	1.84	2.661 (3)	177
O1—H1···N1	0.82	2.02	2.727 (3)	145

Symmetry codes: (i) *x*+1, *y*, *z*.

## References

[bb1] Ahmad, T., Zia-ur-Rehman, M., Siddiqui, H. L., Mahmud, S. & Parvez, M. (2010). *Acta Cryst.* E**66**, o1022. 10.1107/S1600536810011864 PMC2979035 21579086

[bb2] Ajani, O. O., Obafemi, C. A., Nwinyi, O. C. & Akinpelu, D. A. (2010). *Bioorg. Med. Chem.* **18**, 214–221. 10.1016/j.bmc.2009.10.064 19948407

[bb3] Angelusiu, M. V., Barbuceanu, S. F., Draghici, C. & Almajan, G. L. (2010). *Eur. J. Med. Chem.* **45**, 2055–2062. 10.1016/j.ejmech.2010.01.033 20133023

[bb4] Bernstein, J., Davis, R. E., Shimoni, L. & Chang, N.-L. (1995). *Angew. Chem. Int. Ed. Engl.* **34**, 1555–1573.

[bb5] Bruker (2001). *SADABS* Bruker AXS Inc., Madison, Wisconsin, USA.

[bb6] Bruker (2007). *SMART* and *SAINT* Bruker AXS Inc., Madison, Wisconsin, USA.

[bb7] Huang, H.-T. & Wu, H.-Y. (2010). *Acta Cryst.* E**66**, o2729–o2730. 10.1107/S1600536810038857 PMC3009357 21588939

[bb8] Ji, X.-H. & Lu, J.-F. (2010). *Acta Cryst.* E**66**, o1514. 10.1107/S1600536810019914 PMC2979514 21579573

[bb9] Khaledi, H., Alhadi, A. A., Mohd Ali, H., Robinson, W. T. & Abdulla, M. A. (2010). *Acta Cryst.* E**66**, o105–o106. 10.1107/S1600536809052465 PMC2980105 21579996

[bb10] Sheldrick, G. M. (2008). *Acta Cryst.* A**64**, 112–122. 10.1107/S0108767307043930 18156677

[bb11] Singh, V. P. & Singh, S. (2010). *Acta Cryst.* E**66**, o1172. 10.1107/S1600536810010937 PMC2979270 21579213

[bb12] Zhang, Y.-H., Zhang, L., Liu, L., Guo, J.-X., Wu, D.-L., Xu, G.-C., Wang, X.-H. & Jia, D.-Z. (2010). *Inorg. Chim. Acta*, **363**, 289–293.

[bb13] Zhou, C.-S. & Yang, T. (2010). *Acta Cryst.* E**66**, o290. 10.1107/S1600536809055330 PMC2979746 21579725

